# *BRCA1/2* Testing Landscape in Ovarian Cancer: A Nationwide, Real-World Data Study

**DOI:** 10.3390/cancers16091682

**Published:** 2024-04-26

**Authors:** Lieke Lanjouw, Joost Bart, Marian J. E. Mourits, Stefan M. Willems, Annemieke H. van der Hout, Arja ter Elst, Geertruida H. de Bock

**Affiliations:** 1 Department of Epidemiology, University Medical Center Groningen, University of Groningen, 9700 RB Groningen, The Netherlands; 2 Department of Pathology and Medical Biology, University Medical Center Groningen, University of Groningen, 9700 RB Groningen, The Netherlands; 3 Department of Obstetrics and Gynecology, University Medical Center Groningen, University of Groningen, 9700 RB Groningen, The Netherlands; 4 Department of Genetics, University Medical Center Groningen, University of Groningen, 9700 RB Groningen, The Netherlands

**Keywords:** *BRCA1/2* pathogenic variants, ovarian carcinoma, tumor test, next-generation sequencing, real-world data

## Abstract

**Simple Summary:**

Nowadays, tumor tests to analyze DNA in tumor cells from epithelial tubal/ovarian cancers (EOCs) are performed in many centers to detect tumor pathogenic variants (TPVs) in the *BRCA1/2* genes. Information on the presence of these TPVs guides treatment options and further genetic testing in patients and relatives. However, there is no standardization of testing procedures, and information about how testing is performed is limited. Therefore, we described how *BRCA1/2* tumor testing is performed in 999 EOC patients in the Netherlands in 2019 using real-world clinical data. Tumor tests were performed for 502 patients (50.2%) and TPVs were detected in 14.7% of the tests. This study shows that there is variability in the execution of *BRCA1/2* tumor tests, but there were no indications for quality differences. Adequate reporting and quality monitors are essential to ensure that all centers perform reliable tumor tests to ultimately identify all patients with *BRCA1/2* TPVs.

**Abstract:**

Analyzing *BRCA1/2* tumor pathogenic variants (TPVs) in epithelial tubal/ovarian cancers (EOCs) has become an essential part of the diagnostic workflow in many centers to guide treatment options and genetic cascade testing. However, there is no standardization of testing procedures, including techniques, gene assays, or sequencers used, and data on the execution of tumor tests remains scarce. Therefore, we evaluated characteristics of *BRCA1/2* tumor testing in advanced-stage EOC with real-world national data. Pathology reports of patients diagnosed with EOC in 2019 in the Netherlands were obtained from the Dutch Pathology Registry (PALGA), and data regarding histological subtype and *BRCA1/2* tumor tests were extracted. A total of 999 patients with advanced-stage EOC were included. Tumor tests were performed for 502 patients (50.2%) and *BRCA1/2* TPVs were detected in 14.7%. Of all tests, 48.6% used hybrid capture techniques and 26.5% used PCR-based techniques. More than half of the tests (55.0%) analyzed other genes in addition to *BRCA1/2*. Overall, this study highlights the heterogeneity in the execution of *BRCA1/2* tumor tests. Despite a lack of evidence of quality differences, we emphasize that adequate reporting and internal and external quality monitors are essential for the high-quality implementation and execution of reliable *BRCA1/2* tumor testing, which is crucial for identifying all patients with *BRCA1/2* TPVs.

## 1. Introduction

In recent years, testing on *BRCA1/2* pathogenic variants (PVs) in patients diagnosed with epithelial tubal/ovarian cancer (EOC) has become increasingly important. While germline testing for *BRCA1/2* PVs has been available for EOC patients in most medical centers for over a decade, the introduction of the poly (ADP-ribose) polymerase inhibitor (PARPi) therapy has created the need to also identify patients with somatic PVs, as tumors with *BRCA1/2* PVs (somatic and germline) exhibit superior sensitivity towards this therapy [1,2,3,4,5]. Clinically meaningful overall survival benefits have been reported in EOC patients with a germline or somatic *BRCA1/2* PV receiving the PARPi olaparib for two years; 67.0% of the patients receiving olaparib were alive after seven years, versus 46.5% of the patients in the placebo arm [1].

The American Society of Clinical Oncology (ASCO) and the European Society of Medical Oncology (ESMO) recommend performing both somatic and germline testing in EOC patients [6,7]. This identifies patients with genetic predisposition to the disease, which could have clinical implications for the family members of the patient, but also identifies those patients who are likely to benefit from PARPi therapy. To efficiently perform both tests, many centers analyze DNA from tumor samples by first using next-generation sequencing (NGS) and subsequently analyzing germline pathogenic variants (GPVs) only in those with a PV in the tumor (also referred to as tumor PV (TPV)). Patients with a positive family history and/or no or inconclusive tumor test results are also eligible for germline testing. This sequential workflow reduces the number of referrals for genetic counseling and germline testing, as well as the associated patient burden, and is considered cost-effective [8,9,10].

In the Netherlands, this tumor-first workflow is fully implemented in specialized centers. However, there is no standardization of testing procedures, including the techniques, gene assays, or sequence machines used for the analyses. In addition to a lack of national guidelines on testing procedures, data on the performance of the tumor tests, as well as test outcomes, throughout the Netherlands remain scarce. For these reasons, we evaluated the execution of *BRCA1/2* TPV testing in the Netherlands with real-world data from 2019 and provided insight into the number of *BRCA1/2* TPVs detected, and the techniques used in Dutch testing centers. 

## 2. Materials and Methods

Patients diagnosed with EOC in 2019 in the Netherlands were identified with the help of the nationwide network and registry of histo- and cytopathology in the Netherlands (PALGA) [11]. The PALGA database contains excerpts of all pathology reports from pathology departments in the Netherlands and has had full national coverage since 1991. The pathology laboratories in the Netherlands are ISO-15189-certified and the quality of the accredited laboratories is evaluated through various ways, including internal and external audits as well as interlaboratory quality comparisons [12].

Anonymous pathology reports regarding the execution of *BRCA* tumor tests were retrieved from PALGA for all patients diagnosed with EOC in 2019 in the Netherlands. These pathology reports were subsequently linked to data from the Netherlands Cancer Registry [13] to obtain the FIGO stage for each patient. Patients were excluded if they were diagnosed with FIGO stage I or II EOC, as these patients had no indication for adjuvant PARPi therapy [14].

For all included patients, data regarding the histological subtype of the tumor and the *BRCA1/2* tumor NGS analyses were obtained from the pathology reports. These data include, amongst other variables, the following: tumor NGS analysis performed (yes/no); tumor NGS results; technique used; and genes analyzed. Additionally, it was checked whether the tumor test was complemented with a *BRCA1* multiplex ligation-dependent probe amplification (MLPA) analysis (yes/no) and, if yes, the MLPA test result was collected (*BRCA1* PV: yes/no). Information on the detection of variants of unknown clinical significance (VUS) was also collected when reported in the pathology reports. Dutch pathology laboratories follow national guidelines for establishing the classification and relevance of detected variants, which include close collaboration with the genetics departments of medical centers [15].

In the case of a detected *BRCA1/2* TPV in the EOC of an ambiguous or unspecified histological subtype (e.g., carcinoma not otherwise specified (NOS)), an expert pathologist reviewed the pathology reports and further classified the tumor, if possible, based on the information from the corresponding pathology reports and according to the World Health Organization’s classification of the female genital tumors of 2020 [16].

The following endpoints were analyzed in this cohort of advanced-stage EOC patients: (1) the number of diagnosed EOC by histological subtype; (2) the prevalence of *BRCA1/2* TPVs and VUS by histological subtype; (3) the (reporting of) techniques and platforms used in *BRCA1/2* tumor NGS analyses, including the specific genes analyzed; and (4) the lead times of the *BRCA1/2* tumor analyses.

Data were reported as frequencies and percentages and lead times as mean, standard deviation and minimum and maximum values. Information on the type of technique used for target enrichment was collected and classified as hybrid capture techniques or polymerase chain reaction (PCR)-based amplicon techniques. The number of TPVs detected by *BRCA1* MLPA analysis was reported separately from those detected by NGS. The number of *BRCA1/2* TPVs was compared between the hybrid capture and PCR-based techniques using the Chi-square exact test. The distribution of the histological subtype was also compared between the hybrid capture and PCR-based techniques using Fisher’s exact test. The lead time of the *BRCA1/2* tumor analyses was defined as the number of days between the date of the receival of tumor material in pathology centers and the reported date of the tumor test results. The lead time could only be calculated when both dates were reported.

## 3. Results

**Figure 1:**
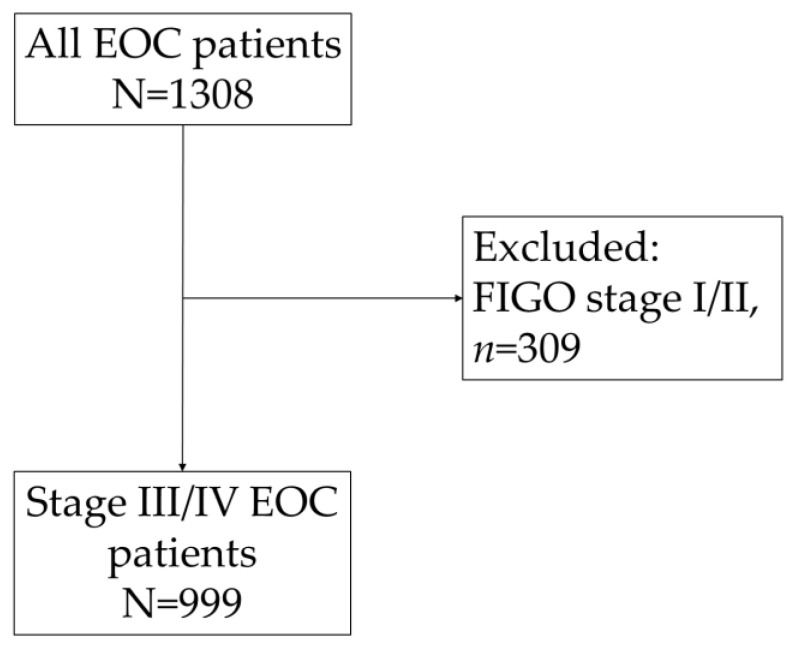
Flowchart of stage III/IV EOC patient selection.

The PALGA search identified 1308 women who were diagnosed with EOC in 2019 in The Netherlands (Figure 1). After the exclusion of patients with early-stage disease (FIGO I/II) (*n* = 309; 23.6%), a total of 999 EOC patients were included. Most EOC patients were diagnosed with high-grade serous carcinoma (*n* = 682; 68.3%), followed by carcinoma NOS (*n* = 65; 6.5%) and low-grade serous carcinoma (*n* = 46; 4.6%) (Table 1). The histological subtype was not reported in the pathology report for 5.8% of the patients.

*BRCA1/2* tumor NGS analyses were performed for 502 patients (50.3%), and a total of 62 TPVs were detected (12.4% of all NGS analyses); 31 TPVs in *BRCA1* and 31 TPVs in *BRCA2* (Table 2). A complementary *BRCA1* MLPA analysis was performed for 344 patients (34.4%) and it detected an additional 12 *BRCA1* TPVs (3.5% of all MLPA analyses). Combining the TPVs detected through NGS and MLPA analyses (*n* = 74), most *BRCA1/2* TPVs were detected in high-grade serous carcinoma (*n* = 67; 90.5%) (Supplementary Table S1). The remaining seven TPVs were detected in low-grade serous carcinoma (*n* = 1; 1.4%), endometrioid carcinoma (*n* = 1; 1.4%), clear cell carcinoma (*n* = 1; 1.4%), carcinosarcoma (*n* = 3; 4.1%) and carcinoma NOS (*n* = 1; 1.4%). In addition, the detection of six VUS was reported in the pathology reports. Five VUS (83.3% of all VUS) were reported in high-grade serous carcinoma and one VUS (16.7%) in endometrioid carcinoma. Caution must be taken when interpreting the number of VUS reported in this study, since reporting a detected VUS in the pathology report is not universally adopted by all the testing centers. 

**Figure 2:**
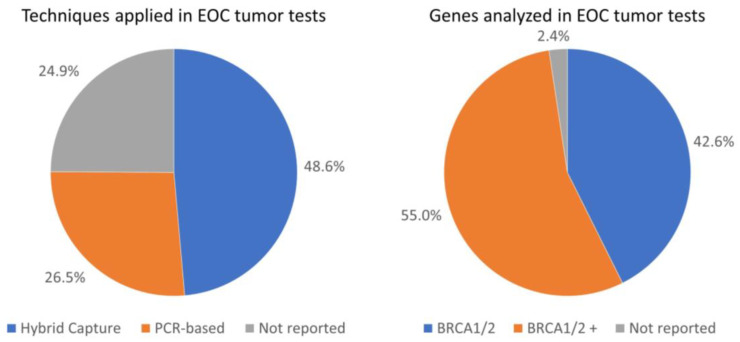
Distribution of the techniques applied and genes analyzed in EOC tumor tests.

Figure 2 visualizes the distribution of the different techniques applied for the target enrichment and the genes analyzed in the EOC tumor tests. Of the 502 *BRCA1/2* tumor analyses performed in our cohort, a total of 244 analyses (48.6%) were performed using the hybrid capture technique and 133 analyses (26.5%) using the PCR-based technique (Figure 2). Information on the target enrichment technique applied was missing for a substantial proportion of the performed *BRCA1/2* tumor analyses (*n* = 125, 24.9%). The proportion of *BRCA1/2* TPVs detected did not significantly differ between the hybrid capture and PCR-based techniques (12.3% and 21.1%, respectively; *p*-value = 0.078), neither did the distribution of histological subtypes between the hybrid capture and PCR-based techniques (*p*-value = 0.882) (Supplementary Table S2). Of all NGS analyses, 42.6% analyzed exclusively the *BRCA1/2* genes, and 55.0% used a more comprehensive panel, also including genes other than *BRCA1/2* (referred to as *BRCA1/2*+ in Figure 2). For 2.4% of all NGS analyses, the specific genes analyzed were not reported. 

All tests performed using the hybrid capture technique used the single-molecule molecular inversion probes (smMIPs) method (*n* = 244) (Table 3). A total of four different assays were used for the PCR-based techniques: custom Ampliseq BRCAv5 assay (Thermo Fisher Scientific Inc., Waltham, MA, USA) (*n* = 59; 44.4%); BRCA Tumor MASTR Plus assay (Multiplicom/Agilent Technologies, Inc., Santa Clara, CA, USA) (*n* = 62; 46.6%); Oncomine BRCA Research assay (Thermo Fisher Scientific Inc., Waltham, MA, USA) (*n* = 11; 8.3%) and SureMASTR HRR assay (Agilent Technologies, Inc., Santa Clara, CA, USA) (*n* = 1; 0.8%). The assays used and genes analyzed in the analyses not reporting target enrichment techniques are reported in Supplementary Table S3.

More than half of the *BRCA1/2* tumor analyses were performed on an Illumina platform (Illumina, Inc., San Diego, CA, USA) (*n* = 262; 52.2%), 15.1% on an Ion Torrent platform (Thermo Fisher Scientific Inc., Waltham, MA, USA), and in more than 30% of the *BRCA1/2* tumor analyses, the platform used was not specified (*n* = 156; 31.1%) (Supplementary Table S4). Lead times were analyzed for the cases where the dates of the receival of the tumor material and test results were reported in the pathology report (*n* = 376). The mean lead time was 38.3 days (SD = 64.2 days), ranging from 0 days to 525 days (Supplementary Table S5).

## 4. Discussion

The current study provides insight into the execution and outcomes of *BRCA1/2* tumor analyses in patients diagnosed with advanced-stage EOC in 2019 in the Netherlands. Of the 999 advanced stage EOC patients included in this study, *BRCA1/2* tumor NGS analyses were performed for 502 patients (50.3%). Most importantly, this study shows that substantial variety exists in the execution of tumor analyses in EOC regarding the techniques and assays used, and the (types of) genes analyzed.

To the best of our knowledge, this study is the first to provide insight into the nationwide landscape of *BRCA1/2* tumor testing in EOC. Besides providing insight into the applied techniques, assays and analyzed genes, this study also highlights the lack of uniform reporting in pathology reports, despite high-quality centralized care and the utilization of a national pathology registration database. A great proportion of the pathology reports lacked information on the techniques and assays used for the analyses, the analyzed genes, and dates of, for example, the test results. For this reason, lead times could only be analyzed for a subset of 376 tests. Importantly, lead time is included in the criteria of the national quality control standards that are currently being implemented [17]. Inadequate reporting limited quality assessment in the current study and, more importantly, could have clinical implications for patients, as it limits the exchange of diagnostic information between clinicians and the tailoring of a patient’s treatment. Considering the increased sensitivity of patients with *BRCA1/2* TPVs towards PARPi therapy, and the possible heredity of the disease, the complete reporting of these analyses is extremely important. Fortunately, studies show that pathology reporting in oncology is changing from a narrative approach to standardized synoptic reporting, leading to a significantly increased completeness of the pathology reports [18,19,20].

A complementary *BRCA1* MLPA analysis was performed for 344 patients. In these patients, the MLPA analysis detected an additional 3.5% of *BRCA1* TPVs. MLPA analyses are generally applied to detect large rearrangements, such as the deletions or duplications of complete exons or multiple exons. While NGS is considered a reliable tool to detect point mutations, which comprise most *BRCA1/2* PVs, the NGS is less sensitive to detecting large rearrangements. The large arrangements particularly occur in the *BRCA1* gene and are known to be more prevalent in certain populations, including the Dutch population [21,22,23,24]. Conducting NGS with a complementary MLPA analysis is frequently regarded as offering a comprehensive evaluation of potential genomic changes in the *BRCA1/2* genes, whereas when solely NGS is employed, potential TPVs may be missed. Estimates of the prevalence of large genomic rearrangements in *BRCA1* in EOC specifically remain limited, which makes it challenging to estimate the number of TPVs missed when not performing an MLPA analysis alongside the NGS. A Slovakian study performed MLPA analyses in 39 tumor samples of high-grade serous ovarian cancer and detected one pathogenic *BRCA1* deletion (2.6%) [25]. Pathogenic large rearrangements were also analyzed in 20,000 ovarian tumors with NGS and were detected in 0.7% of the cases, which reflected a total of 6.3% of all *BRCA1/2* TPVs detected in the cohort [26]. This relatively low percentage could be explained by the lower sensitivity of NGS in detecting large deletions and duplications and may, therefore, underestimate the prevalence of these large rearrangements. Furthermore, the presence of founder mutations, as established in *BRCA1* in the Netherlands [24], increases the number of TPVs to be detected by MLPA analysis and should be considered when making direct comparisons. 

The proportion of *BRCA1/2* TPVs detected did not significantly differ between the hybrid capture technique, which constituted solely smMIP-based assays [27], and the PCR-based techniques and does not, therefore, indicate significant performance differences between the techniques regarding *BRCA1/2* TPV yield. It must be noted that for a thorough comparison of *BRCA1/2* yield in hybrid capture versus the PCR-based technique, a more diverse inclusion of tests using the hybrid capture technique is preferred. Few studies have compared the overall performance of hybrid capture and PCR-based approaches in detecting PVs. A better overall performance was reported for the hybrid capture technique in detecting *BRCA1/2* PVs from formalin-fixed paraffin-embedded (FFPE) EOC tumor samples compared to the PCR-based technique [28], and similar findings were reported in a study assessing the detection of actionable mutations in lymphoma [29]. In general, these studies linked the PCR-based technique to a lower sensitivity due to amplicon dropout and insufficient coverage. On the other hand, PCR-based techniques are also reported to be suitable for the accurate detection of *BRCA1/2* PVs [30]. Moreover, it requires lower quality and quantity of DNA and is significantly less time consuming, which are important parameters for a laboratory to consider when choosing between methods [31,32,33]. The results of the current study do not show quality differences between the techniques, thereby justifying the use of both techniques in *BRCA1/2* TPV detection. The selection of methods, genes and sequence machines is often carried out by individual laboratories and is generally based on several aspects, including reliability, lead times and costs. The latter could not be evaluated in the current study since this information was not available. In the Netherlands, pathology laboratories are free to choose techniques given the technique is validated, and the national quality control standard for molecular diagnostics ensures that these techniques meet high-quality criteria [17]. This subsequently guarantees high-quality diagnostics and care for all patients. 

A total of 74 *BRCA1/2* TPVs were detected in this cohort of Dutch EOC patients (14.7% of all tests), of which 90.5% were detected in high-grade serous carcinoma. Our overall proportion of *BRCA1/2* TPVs in EOC, unselected for histotype, is similar to the 13% proportion we reported previously in a Dutch multi-center study that included a consecutive series of EOC patients [34]. The proportion is slightly lower compared to the 16.7% proportion in EOC reported by another Dutch study, which included a complementary MLPA analysis for all cases, and also lower compared to the 19% proportion reported in the United States [8,35]. 

This study shows that EOC tumor tests for *BRCA1/2* TPV detection were already performed for 50% of all patients before this was officially recommended by national and international guidelines [6,7,36]. Currently, tumor testing is implemented nationwide; testing is centralized mostly in academic hospitals, and comprehensive gene panels are more frequently applied. It should be noted that the *BRCA1/2* tumor test rate of 50.3% reported in this study does not imply that only half of all patients received *BRCA1/2* testing. The timeframe analyzed here was before national guidelines recommended tumor testing in EOC; therefore, it is likely that medical centers followed former guidelines and referred patients for genetic counseling and germline testing instead [37].

This study has several strengths and limitations. Strengths include the analysis of real-world clinical data with full nationwide coverage of all EOC pathology reports. Linking the data from narrative pathology reports to clinical characteristics such as the FIGO stage allowed us to tailor this evaluation to the population of interest, namely FIGO stage III/IV patients. Nevertheless, data requests from national registries, such as PALGA, are subject to prespecified timeframes and the possibility exists that tumor tests were requested beyond this timeframe for the patients in our population. This may have led to an underestimation of the proportion of EOC patients who received a tumor test. Moreover, data collection for the current study entirely depended on the data reported in the pathology reports. This limited the possibility to evaluate the additional technical aspects of the execution of the tumor tests and restricted the evaluation to the endpoints reported in the current study. Finally, this study analyzed the execution of tumor tests before the full completion of the nationwide implementation of the tumor testing; therefore, it is likely that not all the centers that are currently performing tumor testing were included. Repeating our analyses with the data obtained after the full completion of the national implementation and comparing that data to the results reported in this study can provide valuable insights into the changes in the execution of tumor tests in EOC over time.

## 5. Conclusions

This study highlights the heterogeneity in the execution of EOC tumor testing in the Netherlands in 2019 despite the centralization of testing in specialized centers. The findings of this study are not indicative of any quality differences between the techniques used. Furthermore, nationally implemented quality control standards ensure the high-quality implementation of reliable *BRCA1/2* tumor testing. This is crucial for identifying all patients with *BRCA1/2* TPVs to provide high-quality care, as well as for guiding genetic cascade testing to ultimately prevent cancer in unaffected relatives with *BRCA1/2* GPVs.

## Figures and Tables

**Table 1:** Number of EOC diagnoses by histological subtype in the Netherlands in 2019.

Histological Subtypes	Stage III/IV EOC Patients N = 999 *n* (%)
High-grade serous	682 (68.3)
Low-grade serous	46 (4.6)
Endometrioid	12 (1.2)
Clear cell	23 (2.3)
Mucinous	11 (1.1)
Carcinosarcoma	27 (2.7)
Carcinoma NOS	65 (6.5)
Other ^1^	75 (7.5)
Unknown	58 (5.8)

^1^ Including: Malignant Brenner tumor, mixed-type histology and undifferentiated carcinoma. Abbreviations: EOC, epithelial ovarian cancer; NOS, not otherwise specified.

**Table 2:** Number of *BRCA1/2* tumor tests performed and the prevalence of *BRCA1/2* TPVs and VUS.

Outcomes of *BRCA1/2* Tumor Analyses	Total *n* (%) ^1^
*BRCA1/2* tumor NGS performed ^2^	502 (50.3)
*BRCA1/2* TPV	62 (12.4)
*BRCA1*	31 (6.2)
*BRCA2*	31 (6.2)
*BRCA1/2* VUS ^3^	6 (1.2)
*BRCA1*	4 (0.8)
*BRCA2*	2 (0.4)
Complementary *BRCA1* MLPA	344 (34.4)
*BRCA1* TPV	12 (3.5)

^1^ Percentages for TPVs and VUS are calculated using the number of tests (NGS or MLPA) as the denominator. ^2^ All analyses were performed using DNA isolated from formalin-fixed paraffin-embedded (FFPE) tumor tissue. ^3^ Detection of VUS is not routinely reported by all testing centers. Abbreviations: MLPA, Multiplex Ligation-dependent Probe Amplification; TPV, tumor pathogenic variants; VUS, variance of unknown significance.

**Table 3:** Assays used and genes tested in epithelial ovarian tumor analyses using hybrid capture (*n* = 244) or PCR-based techniques (*n* = 133).

Assays	Genes	*n* (%) ^1^
Hybrid Capture Technique		
Custom smMIP-based assay		244 (100.0)
	*BRCA1/2*	85 (34.8)
	*BRCA1/2*, *RAD51C/D*, *BRIP1*	159 (65.2)
PCR-based Technique		
Custom Ampliseq BRCAv5 assay		59 (44.4)
	*BRCA1/2*	1 (1.7)
	*BRCA1/2*, *ATM*, *BARD1*, *CDK12*, *CHEK1/2*, *FANCL*, *PALB2*, *PPP2R2A*, *RAD51B/C/D*, *RAD54L*	56 (94.9)
	*BRCA1/2*, *ATM*, *BARD1*, *CDK12*, *CHEK1/2*, *FANCL*, *PALB2*, *PPP2R2A*, *RAD51B/C/D*, *RAD54L*, *RIF1*, *TP53*, *TP53BP1*, *WRN*, *XRCC2/3*	1 (1.7)
	Genes not reported	1 (1.7)
BRCA Tumor MASTR Plus assay		62 (46.6)
	*BRCA1/2*	60 (96.8)
	*ATM*, *ATR*, *BAP1*, *BARD1*, *BLM*, *BRCA1/2*, *BRIP1*, *CDK12*, *CHEK1/2*, *FANCA/C/D2/E/F/L*, *MAD2L2*, *MRE11A*, *NBN*, *PALB2*, *PPP2R2A*, *RAD51B/C/D*, *RAD54L*, *RIF1*, *TP53*, *TP53BP1*, *WRN*, *XRCC2/3*	2 (3.2)
Oncomine BRCA Research assay		11 (8.3)
	*BRCA1/2*	11 (100.0)
SureMASTR HRR assay		1 (0.8)
	*BRCA1/2*, *ATM*, *CHEK2*, *PALB2*, *RAD51C/D*, *BRIP1*	1 (100.0)

^1^ The denominator used for calculating percentages for assays is the type of technique applied; the denominator used for calculating percentages for genes is the assay used. Abbreviations: smMIP, single-molecule molecular inversion probe; PCR, polymerase chain reaction.

## Data Availability

The data presented in this study are available on request from the corresponding author.

## Supplementary Materials

The following supporting information can be downloaded at: https://www.mdpi.com/article/10.3390/cancers16091682/s1, Table S1: Prevalence of *BRCA1/2* TPVs by the histological subtype of EOC. Table S2: Prevalence of *BRCA1/2* TPVs and histological subtypes for analyses reporting hybrid capture or PCR-based techniques. Table S3: Assays used and genes analyzed in analyses not reporting target enrichment techniques. Table S4: Type of platforms used for *BRCA1/2* tumor tests. Table S5: Lead times for *BRCA1/2* tumor tests, reported in days.
