# Supplementary material for: BRCA1/2 Testing Landscape in Ovarian Cancer: A Nationwide, Real-World Data Study
Source: Cancers (Basel). 2024 Apr 26;16(9):1682. doi: 10.3390/cancers16091682 (PMC11083399; doi:10.3390/cancers16091682)
Supplement: Supplementary file 1 [file cancers-16-01682-s001.zip › cancers-2964841-supplementary.pdf]

Supplementary Materials

Table S1. Prevalence of *BRCA1/2* TPVs by the histological subtype of EOC.

	***BRCA1*/2 TPV** *n (%)*	***BRCA1*/2 VUS**[1] *n (%)*
***BRCA1*/2 variants detected**	74 (100.0)	6 (100.0)
High-grade serous	67 (90.5)	5 (83.3)
Low-grade serous	1 (1.4)	.
Endometrioid	1 (1.4)	1 (16.7)
Clear cell	1 (1.4)	.
Mucinous	.	.
Carcinosarcoma	3 (4.1)	.
Carcinoma NOS	1 (1.4)	.

[1] VUS is not always reported. Abbreviations: TPV, tumor pathogenic variant; VUS, variance of unknown significance; NOS, not otherwise specified.

Table S2. Prevalence of *BRCA1/2* TPVs and histological subtypes for analyses reporting hybrid capture or PCR-based techniques.

	**Total** *n (%)*	**Hybrid capture** *n (%)*	**PCR-based** *n (%)*	**P-value**
BRCA1/2 tumor tests performed	502 (100.0)	244 (100.0)	133 (100.0)	
BRCA1/2 TPVs	74 (14.7)	33 (12.3)	28 (21.1)	0.078[1]
BRCA1	43 (8.6)	17 (7.0)	18 (13.5)	
BRCA2	31 (6.2)	16 (6.6)	10 (7.5)	
Histological subtype[2]				
High-grade serous	311 (82.5)	201 (82.4)	110 (82.7)	0.882[3]
Low-grade serous	13 (3.4)	8 (3.3)	5 (3.8)	
Endometrioid	4 (1.1)	2 (0.8)	2 (1.5)	
Clear cell	15 (4.0)	12 (4.9)	3 (2.3)	
Mucinous	3 (0.8)	2 (0.8)	1 (0.8)	
Carcinosarcoma	9 (2.4)	6 (2.5)	3 (2.3)	
Carcinoma NOS	7 (1.9)	4 (1.6)	3 (2.3)	
Other[3]	10 (2.7)	5 (2.0)	5 (3.8)	
Unknown	5 (1.6)	4 (1.6)	1 (0.8)	

[1] Chi-square exact test comparing number of tests detecting *BRCA1/2* PVs for hybrid capture versus PCR-based techniques. [2] Reported for hybrid capture and PCR-based techniques only. [3] Fisher's exact test comparing distribution of histological subtypes for hybrid capture versus PCR-based techniques. [3] Including: Malignant Brenner tumor, mixed type histology and undifferentiated carcinoma. Abbreviations: PCR, polymerase chain reaction; TPV, tumor pathogenic variant; NOS, not otherwise specified.

Table S3. Assays used and genes analyzed in analyses not reporting target enrichment techniques (*n*=125).

Assays and genes		n (%)*
***BRCA1*/2v2**		19 (15.2)
	BRCA1/2	2 (10.5)
	BRCA1/2, CHEK2, TP53, FOXL2	2 (10.5)
	BRCA1/2, ATM, BARD1, BRIP1, CDK12, CHEK1/2, FANCL, PALB2, PPP2R2A, RAD51B/C/D, RAD54L	5 (26.3)
	ATM, ATR, BAP1, BARD1, BRCA1/2, BRIP1, CDK12, CHEK1/2, FANCA/C/E/L, FAND2, MAD2L2, MRE11A, NBN, PALB2, PPP2R2A, RAD51B/C/D, RAD54L, RIF1, TP53, TP53BP1, WRN, XRCC2/3	1 (5.3)
	ATM, ATR, BAP1, BARD1, BLM, BRCA1/2, BRIP1, CDK12, CHEK1/2, FANCA/C/D2/E/F/L, MAD2L2, MRE11A, NBN, PALB2, PPP2R2A, RAD51B/C/D, RAD54L, RIF1, TP53, TP53BP1, WRN, XRCC2, XRCC	1 (5.3)
	Unknown genes	8 (42.1)
**HRDv2**		15 (12.0)
	ATM, ATR, BAP1, BARD1, BLM, BRCA1/2, BRIP1, CDK12, CHEK1/2, FANCA/C/D2/E/L, MAD2L2, MRE11A, NBN, PALB2, PPP2R2A, RAD51B/C/D, RAD54L, RIF1, TP53, TP53BP1, WRN, XRCC2/3	9 (56.3)
	ATM, ATR, BAP1, BARD1, BLM, BRCA1/21, BRIP1, CDK12, CHEK1/2, FANCA/C/D2/E/F, FANCL, MAD2L2, MRE11A, NBN, PALB2, PPP2R2A, RAD51B/C/D, RAD54L, RIF1, TP53, TP53BP1, WRN, XRCC2/3	5 (31.3)
	ATM, BARD1, BRCA1/2, BRIP1, CDK12, CHEK1/2, FANCL/D2, KRAS, MRE11A, NBN, PALB2, PIK3CA, POLE, POLD1, PPP2R2A, PTEN, RAD50, RAD51, RAD51B/C/D, RAD52, RAD54L, TP53, XRCC2	1 (6.3)
**HRDv1.1**		4 (3.2)
	ATM, ATR, BAP1, BARD1, BLM, BRCA1/2, BRIP1, CDK12, CHEK1/2, FANCA/C/D2/E/L, MD2L2, MRE11A, NBN, PALB2, PPP2R2A, RAD51B/C/D, RAD54L, RIF1, TP53, TP53BP1, WRN, XRCC2/3	
**Unknown assay, and unknown genes**		87 (69.6)

* Denominator used for calculating percentages for assays is the total *n*=125, denominator used for calculating percentages for genes is the assay used.

Table S4. Type of platforms used for *BRCA1/2* tumor tests.

Platforms	Total<br>*n (%)*
***BRCA1/2* tumor tests performed**	502 (100.0)
Illumina	262 (52.2)
Ion Torrent	76 (15.1)
Other	8 (1.6)
Not reported	156 (31.1)

Table S5. Lead times for *BRCA1/2* tumor tests, reported in days.

	Lead time<br>*n=376*
Mean	38.3
SD	64.2
Minimum	0
Maximum	525

Abbreviations: SD, standard deviation.
